# Towards Estimating Arterial Diameter Using Bioimpedance Spectroscopy: A Computational Simulation and Tissue Phantom Analysis

**DOI:** 10.3390/s22134736

**Published:** 2022-06-23

**Authors:** Yang Yu, Gautam Anand, Andrew Lowe, Huiyang Zhang, Anubha Kalra

**Affiliations:** Institute of Biomedical Technologies, Auckland University of Technology, Auckland 1010, New Zealand; gautam.anand@aut.ac.nz (G.A.); andrew.lowe@aut.ac.nz (A.L.); huiyang.zhang@aut.ac.nz (H.Z.); anubha.kalra@aut.ac.nz (A.K.)

**Keywords:** electrical impedance spectroscopy, bio-impedance measurement, forearm, artery diameter, finite element analysis, computational simulation, tissue phantom

## Abstract

This paper improves the accuracy of quantification in the arterial diameter-dependent impedance variance by altering the electrode configuration. The finite element analysis was implemented with a 3D human wrist fragment using ANSYS Electronics Desktop, containing fat, muscle, and a blood-filled radial artery. Then, the skin layer and bones were stepwise added, helping to understand the dielectric response of multi-tissues and blood flow from 1 kHz to 1 MHz, the current distribution throughout the wrist, and the optimisation of electrode configurations for arterial pulse sensing. Moreover, a low-cost wrist phantom was fabricated, containing two components: the surrounding tissue simulant (20 wt % gelatine power and 0.017 M sodium chloride (NaCl) solution) and the blood simulant (0.08 M NaCl solution). The blood-filled artery was constricted using a desktop injection pump, and the impedance change was measured by the Multi-frequency Impedance Analyser (MFIA). The simulation revealed the promising capabilities of band electrodes to generate a more uniform current distribution than the traditional spot electrodes. Both simulation and phantom experimental results indicated that a longer spacing between current-carrying (CC) electrodes with shorter spacing between pick-up (PU) electrodes in the middle could sense a more uniform electric field, engendering a more accurate arterial diameter estimation. This work provided an improved electrode configuration for more accurate arterial diameter estimation from the numerical simulation and tissue phantom perspectives.

## 1. Introduction

**Figure 1:**
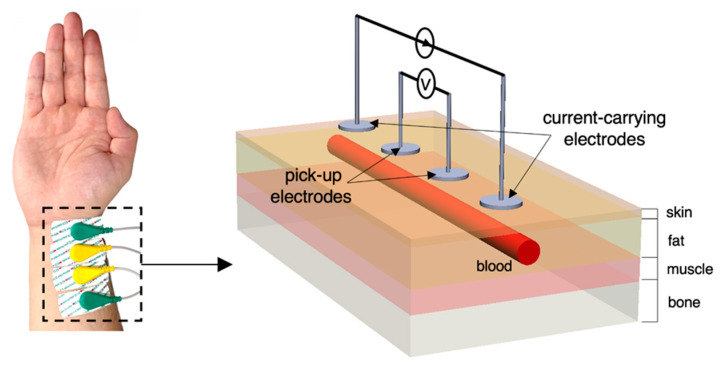
Overview of four-spot electrodes BIM on the human wrist and the equivalent schematic.

In the context of ambulatory hemodynamic monitoring, there has been a booming interest in portable bioimpedance measurement (BIM) or analysis (BIA) for both research and diagnostic purposes [1], which applies the electrodes on limbs such as the forearm near the wrist. It conforms to the current trend of wearable devices for health monitoring, which are easily put on and assessed. The radial artery is a common measured position to detect pulsatile blood due to the thin surrounding tissue layers. For instance, tonometry is a widely used automatic sphygmomanometer to measure arterial blood pressure (BP) from the radial artery [2]. It was demonstrated that the bio-impedance signal measured at radial and ulnar arterial sites showed better signal qualities compared with tibial and carotid arteries [3]. Therefore, wearable BIM on the wrist has been popular due to its capability to sense the blood volume change caused by heart pulsation [4]. When a pulse wave arrives, the amount of blood inside the artery increases and the measured impedance decreases because of the higher conductivity of the blood. Figure 1 illustrates the overall principle of the common four-spot electrodes aligned BIM on the human wrist. Two outer electrodes named current-carrying (CC) electrodes are connected with the alternating current power supply and inject a tiny alternating current (I) into nether tissues from the skin. Two inner electrodes named pick-up (PU) electrodes measure the voltage (V) of a certain distance. The overall measured magnitude impedance ($Z_{overall}$) can be given by:

(1) $$Z_{overall}=\frac{V}{I}$$

The anatomical structure of the human wrist is more symmetrical than the other parts of the body. It can be reasonably assumed to be a uniform cylinder with multiple lumped domains of skin, fat, skeletal muscle, bone, and blood-filled artery. The arterial wall is usually neglected because the wall thickness of the radial artery is too thin compared to the surrounding tissues. In addition to the blood volume changes, it has been widely reported and discussed that the velocity-dependent blood conductivity change is also one importance source for measured impedance changes, specifically, the orientation of red blood cells (RBC), which can trace back to 1968 [5]. For example, the conductivity of flowing blood changed with the shape of velocity profile (i.e., accelerating and decelerating flow) [6]. Recently, several works have been carried out to endeavour to quantify the effects of RBC orientation using mathematical modelling and removed from impedance signals for more accurate arterial diameter estimation [7,8].

Since the last decade, BIM has been widely investigated for the novel cuffless blood BP measurement. For example, BIM on the wrist has been utilised as a distal time reference to determine pulse propagation information for BP estimation [9,10,11,12]. Recently, few studies could estimate continuous BP from a single-channel bio-impedance waveform [13,14].

The sensitivity of the acquired bio-impedance signal often depends on the placement of the electrodes. Several studies have investigated various electrode configurations to obtain a better quality of bio-impedance waveform [9,13,15,16,17]. However, it might lack an understanding of whether the obtained waveforms can accurately represent the responses of measured tissues and pulsatile blood from a mathematical perspective. This might limit our representation of hemodynamic parameters from bio-impedance signals. Different from previous arterial diameter estimation approaches [8,18], this study focuses on the effects of different electrode configurations on the current density and electric field (E-field) distribution within the wrist. The aim is to achieve a reasonably uniform E-field distribution such that the cross-sectional area changes of the blood could be estimated more accurately.

The primary objective of this work is to reach a consensus between simulated/measured resistance values and mathematical modelling of BIM at the human wrist. First, a finite element model (FEM) of the static simplified human wrist segment was constructed using the HFSS design type (ANSYS HFSS) in ANSYS Electronics Desktop (Ansys, Inc., Canonsburg, PA, USA). According to the suggestion from [19], the human wrist 3D model was designed from simple geometry (i.e., electrodes, fat, muscle and blood-filled artery) and then extended stepwise by including other components (i.e., skin and bones). This simulation firstly analysed different dielectric responses of different tissue regions in the β-dispersion. Then after, the pulsatile blood was represented as various arterial diameters. In the meantime, different electrodes’ spacings and types (i.e., spot electrodes and band electrodes) were simulated and compared to observe the promising electrode configuration to generate a nearly uniform E-field. Furthermore, the arterial diameter changes were estimated via the simulated resistance changes.

To validate the above simulations through experiments, a single artery wrist phantom was fabricated to mimic the human forearm near the wrist. A desktop injection pump was applied to alter the blood simulant volume inside the artery. Meanwhile, the impedance signal was measured by the Multi-Frequency Impedance Analyser (MFIA, Zurich Instruments, Zürich, Switzerland). The phantom experiments aimed to substantiate the feasibility of the proposed BIM setup before actual measurement with human body (i.e., frequency range and electrode configurations).

## 2. Materials and Methods

**Figure 2:**
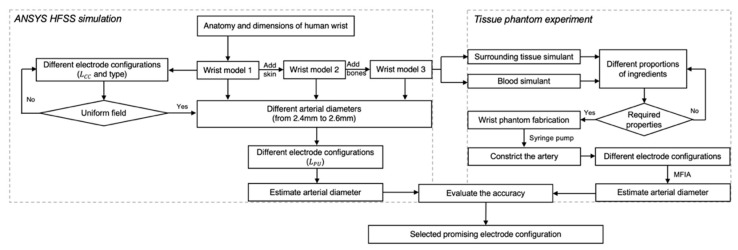
The flowchart of the overall process in this paper, including ANSYS HFSS simulation and tissue-phantom experiments.

The overall methodology for this work is illustrated in Figure 2. Initially, mathematical modelling was performed to analyse the overall contribution of each tissue to the surface BIM. This was followed by simulations performed in ANSYS HFSS. Then, an equivalent wrist phantom was constructed for further verification.

### 2.1. Mathematical Modelling

#### 2.1.1. Wrist Tissue Electrical Modelling

**Figure 3:**
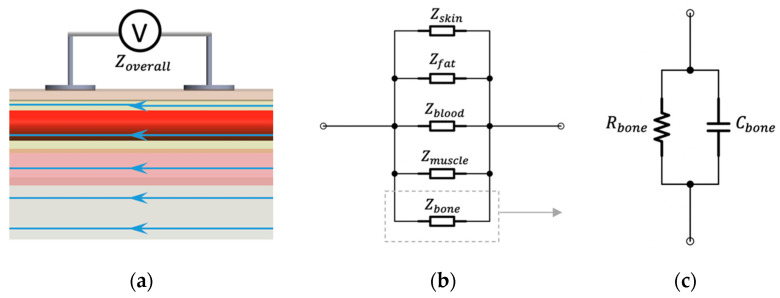
Assumptions for BIM on the wrist: (**a**) ideal parallel current flow; (**b**) equivalent parallel-tissues circuit of measured impedance; (**c**) equivalent parallel RC circuit.

The conduction of current inside the human wrist depends on each tissue’s electrical properties and geometric structures, which makes the equivalent circuit too complicated to represent. To simplify the analysis, we assumed that the current density inside the human wrist was completely uniform, as shown in Figure 3. Moreover, human tissues were regarded as homogeneous with bulk electrical properties. In these conditions, alternating current would be like being injected from both ends of the measured segment, and the various tissues can be regarded as connected in parallel. For tissue components connected in parallel, the inverse overall measured impedance ($Z_{overall}$) equals the summation of the inverses of the component impedances:

(2) $$\frac{1}{Z_{overall}}=\frac{1}{Z_{skin}}+\frac{1}{Z_{fat}}+\frac{1}{Z_{blood}}+\frac{1}{Z_{muscle}}+\frac{1}{Z_{bone}}$$

The β-dispersion (from 1 kHz to 100 MHz) accounts for passive cell membrane capacitance, intracellular organelle membranes and protein molecule response. Human tissues are dielectric materials with both resistance and capacitance in this frequency range. Thus, it is reasonable to describe it as a parallel resistor–capacitor (RC) circuit. For each tissue, the electrical resistance ($R_{tissue}$) can be accurately computed by its electrical conductivity ($\sigma_{tissue}$), cross-sectional area ($A_{tissue}$) and the measured length ($l_{m}$) based on the mentioned assumption.

(3) $$R_{tissue}=\frac{l_{m}}{\sigma_{tissue}A_{tissue}}$$

For the BIM on the wrist, the length of the measured segment can be easily determined by the spacing between PU electrodes ($L_{PU}$). Therefore, in theory, the overall resistance ($R_{overall}$) can be expressed by the conductivity and cross-sectional area of each tissue (i.e., skin (s), blood (b), fat (f), muscle (m), cortical bone (co) and cancellous bone (ca)) and the PU electrode spacing:

(4) $$R_{overall}=\frac{L_{PU}}{\sigma_{s}A_{s}+\sigma_{b}A_{b}+\sigma_{f}A_{f}+\sigma_{m}A_{m}+\sigma_{co}A_{co}+\sigma_{ca}A_{ca}}$$

#### 2.1.2. Modelling Blood Flow in the Wrist

**Figure 4:**
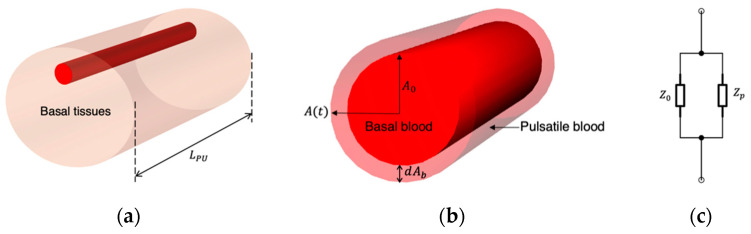
The relationship between the impedance changes and the artery diameter change: (**a**) measured segment of the human wrist; (**b**) cross-sectional area change of the artery; (**c**) equivalent circuit of the pulsatile blood.

Even though researchers have evidenced the effects of RBC orientation on measured impedance changes, it might be challenging to represent such a contribution in an FEM and artificial tissue simulant. Thereby, the blood volume change in the radial artery was regarded as the major contribution to bio-impedance signal change. The modelling of blood flow in the wrist was driven by Nyboer’s theory proposed in 1950 [20] for measuring stroke volume from impedance variation by using an expansible tube. To separate the impedance contribution of pulsatile blood in the artery (AC component) from the overall measured impedance of the wrist (DC component), a parallel combination of the impedance of the pulsatile blood and the static condition of all tissues was assumed, as shown in Figure 4. At any time instant, the overall impedance (Z(t)) during blood flow was modelled as a parallel combination of the basal impedance ($Z_{0}$), which corresponded with the static impedance of all surrounding tissues and static (perfused) blood, and the pulsatile blood ($Z_{p}$), which corresponded with the variable impedance of flowing blood.

(5) $$\frac{1}{Z\left(t\right)}=\frac{1}{Z_{0}}+\frac{1}{Z_{p}}$$

Therefore, the measured change in impedance (dZ) was equal to:

(6) $$dZ=Z\left(t\right)-Z_{0}=\frac{Z_{0}Z_{p}}{Z_{0}+Z_{p}}-Z_{0}=-\frac{Z_{0}{}^{2}}{Z_{0}+Z_{p}}$$

The pulsatile blood $Z_{p}$ could either be positive or negative corresponding to an increment and decrement in the blood volume, respectively. Some previous research mutually converted magnitude impedance and conductivity, which might cause confusion and errors. Strictly speaking, according to Equation (3), only the resistance of the pulsatile blood ($R_{p}$) was related to the conductivity of the blood ($\sigma_{b}$), which can be expressed as:

(7) $$R_{p}=\frac{L_{PU}}{\sigma_{b}\cdot dA_{b}}$$

where $dA_{b}$ was the cross-sectional area changes of the blood ($dA_{b}\neq \;$0). Moreover, Equation (6) can be rewritten by substituting the resistance components:

(8) $$dR=R\left(t\right)-R_{0}=-\frac{R_{0}{}^{2}}{R_{0}+R_{p}}$$

Next, we can substitute Equation (7) into Equation (8), and then, the cross-sectional area changes of the blood could be calculated:

(9) $$dA_{b}=-\frac{L\cdot dR}{\sigma_{b}\cdot R_{0}\cdot R\left(t\right)}$$

where $R_{0}$ was the basal resistance corresponding to the basal tissues and basal cross-sectional area of the blood $A_{0}$. R(t) was the real-time measured resistance, and dR was the difference between basal measured resistance and real-time measured resistance at time t. Suppose we can calibrate the basal inner diameter of the artery through other measurement techniques (e.g., ultrasound image). Then, the real-time inner arterial diameter can be calculated from the cross-sectional area of the blood:

(10) $$d\left(t\right)=2\sqrt{\frac{A\left(t\right)}{\pi }}=2\sqrt{\frac{A_{0}+dA_{b}}{\pi }}$$

The proposed mathematical modelling was based on four main assumptions: (1) the geometry of the artery was a uniform cylindrical plastic tube; (2) the blood was homogeneous with frequency-dependent conductivity (without RBC orientation effects); (3) the volume increment in the blood caused by the pulse was uniform within the measured segment; and (4) the current distribution was parallel to the blood flow.

### 2.2. Computational Simulation

#### 2.2.1. 3D Geometry

The static FEM of a 3D cuboid model (30 mm × 50 mm × 100 mm) of the human forearm segment near the wrist was constructed using the HFSS design type in ANSYS Electronics Desktop (2021 R2). The overall 3D model was composed of two sections, including sensors (i.e., electrodes and cables) and human tissues.

**Figure 5:**
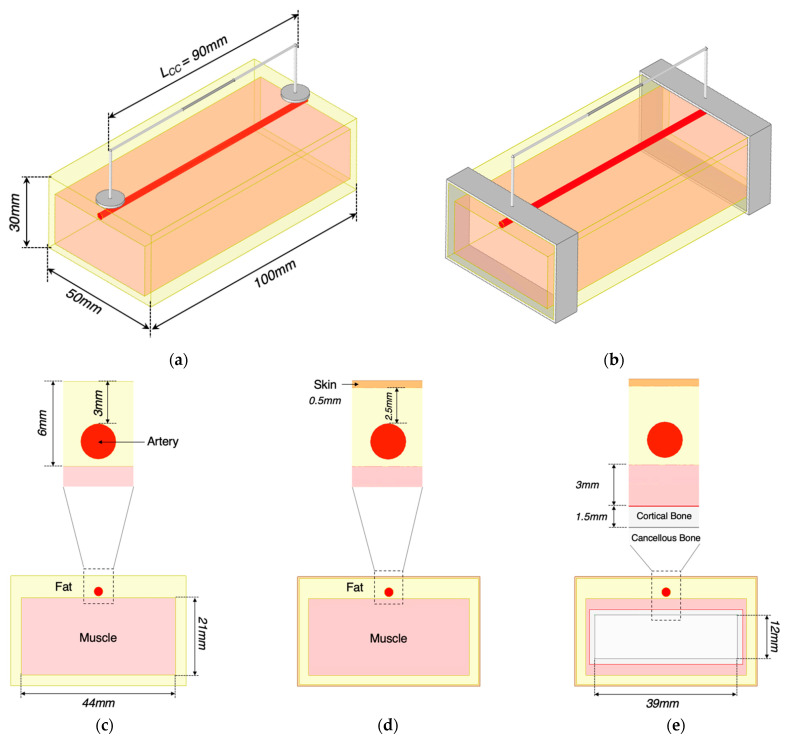
Description of 3D models in ANSYS HFSS: (**a**) conventional spot electrodes setup; (**b**) band electrodes setup; (**c**) a cross-section of the wrist model 1; (**d**) a cross-section of the wrist model 2; (**e**) a cross-section of the wrist model 3.

The simulation setup was investigated through three stages, as described in Table 1 and Figure 5. Each stage corresponded to a combination of different electrode dimensions as well as tissue layers. The dimensions of the models were considered in alignment with several studies and reports in terms of the relative proportions of tissues in the standard human forearm [21], depth and diameter of the human radial artery [22], human forearm anatomy scanned by Magnetic Resonance Imaging (MRI) [23,24,25,26], the ultrasonic imaging of human volar forearm [27], the average diameters of the radial artery and ulnar artery of the population [28,29,30], and human wrist circumference [31]. Additionally, tissues’ properties were considered constant with time; thereby, the bulk conductivity (σ) and relative permittivity ($\varepsilon_{r}$) were assigned to each domain based on [32,33,34].

In this study, the blood volume change in the main artery (without the effects of the RBC orientation) was regarded as the only dominant contribution to bio-impedance signal change acquired at the wrist. Thereby, the time-varying blood flow caused by heart pulsatile was represented as the blood volume change with five different static arterial diameters of 2.4 mm, 2.45 mm, 2.50 mm, 2.55 mm and 2.60 mm based on the actual vasoconstriction and vasodilation range of the human radial artery. The arterial diameter of 2.50 mm was defined as the basal condition, while other variables were used to calculate the increment/decrement in simulated impedance and estimate the contraction/expansion of the blood cross-sectional area. The small pulsatile blood inside other smaller arteries and arterioles during the circulation was neglected.

The typical four-electrode configuration aligned on the radial artery was firstly investigated in this simulation. CC electrodes were modelled as a thin cylinder shape to represent the conventional silver/silver chloride (Ag/AgCl) electrodes. In addition to the spot electrode, the band electrode encircling the whole wrist was also investigated. CC electrodes were placed at different spacings from centre to centre to explore the effects of spacing ($L_{CC}$) on the current distribution, including 90 mm, 70 mm and 50 mm.

#### 2.2.2. Simulation Setup

Curvilinear Elements were applied to the artery domain to represent its curvature geometry accurately. The mesh density increased around the CC electrodes to have minimal influences on the results.

The Current Excitation was applied between two CC electrodes as 1 mA within the safety of medical equipment and prescribed allowable current ranges. The discrete frequency sweep option was chosen. Eleven current injection frequencies were carried out within the frequency range in the β-dispersion (i.e., 1 kHz, 100 kHz, 200 kHz, 300 kHz, 400 kHz, 500 kHz, 600 kHz, 700 kHz, 800 kHz, 900 kHz and 1 MHz,). Furthermore, the whole wrist model was enveloped within a radiation boundary and a vacuum region to simulate the prevention of any electromagnetic interference from an outside source. It could also limit the space of the overall region to ensure the simulation speed.

#### 2.2.3. Data Processing

In this work, three pairs of the location of PU electrodes were investigated, including from 15 to 65 mm, from 25 to 55 mm and from 35 to 45 mm, corresponding to the PU electrodes’ spacings ($L_{PU}$) of 50 mm, 30 mm, and 10 mm, respectively. Unlike the CC electrodes, the PU electrodes were not structured in a specific geometry in the 3D model. A non-model line was drawn between two CC electrodes to export the real (Re(Z)) and imaginary (Im(Z)) parts of the complex impedance.

In this research, the equivalence circuit of human tissues is simplified as a resistor connected in parallel with a capacitor. Thus, the real and imaginary part of impedance were expressed as:

(11) $$Re\left(Z\right)=\frac{R_{s}}{1+{\left(2\pi fR_{s}C_{s}\right)}^{2}}$$

(12) $$Im\left(Z\right)=-\frac{2\pi fR_{s}^{2}C_{s}}{1+{\left(2\pi fR_{s}C_{s}\right)}^{2}}$$

where f was the excitation frequency. The simulated resistance $R_{s}$ was then obtained by solving Equations (11) and (12).

### 2.3. Tissue Phantom Experiment

#### 2.3.1. Phantom Mould Design and Fabrication

**Figure 6:**
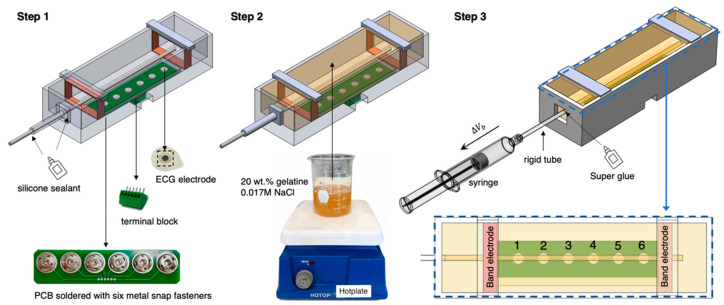
The whole fabrication process of the wrist phantom.

Figure 6 illustrates the whole fabrication process of the wrist phantom. According to Equation (9), the estimation of arterial diameter changes only required the conductivity of blood rather than other tissues (i.e., skin, fat, muscle, and bones). Furthermore, considering the cost and complexity of the phantom fabrication, all other tissues were reasonably considered as one uniform domain based on the equivalent parallel-tissues circuit shown in Figure 3b.

To avoid sacrificing the stable contact between electrodes and the phantom surface, we placed Ag/AgCl dry electrodes (spot electrodes) on an electrode plate and fixed it at the bottom of the mould to ensure a long-term stable attachment during the measurement. First, six metal snap fasteners were soldered on the printed circuit board (PCB) to easily replace the ECG dry electrodes, considering the electrode surface may be oxidised and corroded in contact with the phantom for a long time. Then, a six-pin PCB terminal block (MPT 0.5/5-2.54, Phoenix Contact) was soldered on another side of the PCB to connect to the MFIA. Six pins were individually connected to six ECG dry electrodes through the PCB.

**Figure 7:**
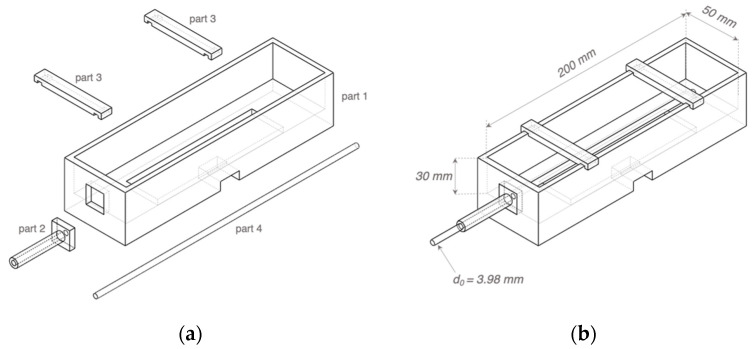
Three-dimensional (3D) schematics of the phantom mould: (**a**) separated 3D printed parts 1–3 and the metal rod part 4; (**b**) the assembled mould.

The phantom mould was designed using SOLIDWORKS 2019 and then 3D printed using the Polyethylene Terephthalate Glycol (PETG) filaments, which showed excellent tolerances to high temperature and corrosion during the phantom fabrication. As shown in Figure 7, the inner width and height of the mould (part 1) remained the same as the simulated 3D wrist model shown in Figure 5, which were 50 mm and 30 mm, respectively. The inner length was 200 mm to leave adequate space for the electrode plate and future operation of the pump. Part 2 was a dismountable component to guide and stabilise the straight metal rod (part 4). The metal rod with a diameter ($d_{0}$) of 3.98 mm was utilised to cast the hollow artery section. The rod diameter was larger than the radial artery diameter simulated in ANSYS HFSS to ensure an easier pumping operation. Furthermore, two pieces of part 3 could clip on part 1 to provide four-sided structures for copper tapes as the band electrodes. After assembling all parts and the electrode plate, a thin silicon insulating layer (Ecoflex^TM^ 00-50) was poured on the electrode plate to cover all other conductive components except the ECG dry electrodes. This step was essential to prevent other conductive components from contacting the phantom.

#### 2.3.2. Selection of Tissue-Mimicking Materials

This project focused on two phantoms/simulants: gel-based surrounding tissue simulant and conductive liquid blood simulant. In the beginning, the selection of suitable tissue-mimicking materials referred to our previous works [35,36].

1. Surrounding tissue simulant

All surrounding tissues were considered as a whole fundamental component, including skin, fat, muscle, and bones. Gelatine was the most common matrix material used in tissue phantoms as the heterogeneous water-soluble mixture of high average molecular weight. The advantages of gelatine-based phantoms were that they could be fabricated at a low cost and preserved in a refrigerator for a long time.

While no prerequisites were considered for mechanical properties of the surrounding tissue phantom, it was ensured that the phantom exhibits adequate rupture strength to maintain the integrity of the artery section during pumping. A positive correlation between Young’s modulus and gel concentration has been found for gelatine in our previous work [35]. The higher gel concentration resulted in increased stiffness. Moreover, our previous investigation [35] demonstrated that higher gelatine concentration showed higher tolerance to deformation (breaking strain). Considering the deformation range of the artery, 20 wt % gelatine powder was selected as the matrix of the surrounding tissue phantom for further experiment in this project.

The conductivity of the surrounding tissue phantom was mimicked to approximate the overall conductivity of simulated skin, fat, muscle, cortical bone, and cancellous bone domains in ANSYS HFSS. The approximate target conductivity of the surrounding tissue phantom was calculated via the cross-section and the individual conductivity of each tissue. Based on the equivalent parallel-tissues electrical modelling, the overall conductivity of surrounding tissues ($\sigma_{st}$) can be determined by:

(13) $$\begin{array}{cc} \sigma_{st}=\frac{l}{R_{st}A_{st}}=\frac{\left(\frac{1}{R_{\mathrm{skin}}}+\frac{1}{R_{\mathrm{fat}}}+\frac{1}{R_{\mathrm{muscle}}}+\frac{1}{R_{\mathrm{cortical}}}+\frac{1}{R_{\mathrm{cancellous}}}\right)l}{A_{\mathrm{st}}}\\ =\frac{\sigma_{\mathrm{skin}}A_{\mathrm{skin}}+\sigma_{\mathrm{fat}}A_{\mathrm{fat}}+\sigma_{\mathrm{muscle}}A_{\mathrm{muscle}}+\sigma_{\mathrm{cortical}}A_{\mathrm{cortical}}+\sigma_{\mathrm{cancellous}}A_{\mathrm{cancellous}}}{A_{\mathrm{skin}}+A_{\mathrm{fat}}+A_{\mathrm{muscle}}+A_{\mathrm{cortical}}+A_{\mathrm{cancellous}}} & \end{array}$$

The calculated overall conductivity of all surrounding tissues was from 0.0993 to 0.1498 S/m between 1 kHz and 1 MHz. The previous results in [35] showed that the gelatine-only samples were much less conductive than the target values, from approximately 0.0164 to 0.0298 S/m between 1 kHz and 1 MHz. Sodium chloride (NaCl) was used to increase conductivity, and testing showed a linear increase in conductivity with the amount of added NaCl. Eventually, a solution of 0.1 wt % NaCl (approximately 0.017 M) in gelatine was selected as the surrounding tissue simulant in this phantom experiment.

2. Blood simulant

**Figure 8:**
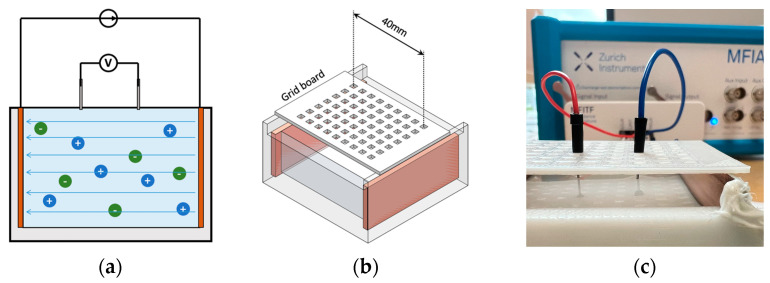
Four-electrode conductivity measurement: (**a**) the principle of the four-electrodes method; (**b**) 3D schematic of the setup; (**c**) experimental setup for NaCl solution samples.

Human blood is a highly conductive tissue with conductivity between 0.70 and 0.82 S/m within 1 kHz and 1 MHz. Due to ease of availability, NaCl solution became the first choice to mimic the target conductivity of blood. First, three different concentrations of NaCl solution (i.e., 0.03 M, 0.05 M and 0.15 M) were prepared by adding the weighted NaCl with 100 mL deionised water and then fully dissolved by stirring for around 2 min using a magnetic stirrer. NaCl samples were prepared shortly before the conductivity measurement at room temperature (22.5 °C). A four-electrode method was employed to measure the conductivity of liquid samples (see Figure 8). The maximum volume of the cuboid liquid container was 50 mL (50 mm ×50 mm × 20 mm). In this low-cost setup, the outer two plate electrodes generated uniform current excitation, and the inner two pin electrodes measured the resulting potential difference. A 3D-printed grid board was placed on the cuboid liquid container. There were seven rows of small holes (with 9 holes in each row and a spacing of 5 mm between each hole) to insert and fix the pin electrodes. The uniformity of the E-field throughout liquid was verified by comparing the measured impedance between each row with the same pin electrode spacing. The measurement setup was connected to the MFIA, and the real and imaginary parts of impedance were measured between 1 kHz and 1 MHz and exported for further data processing.

In this project, tissue simulants were also simplified as parallel circuits with a resistor and a capacitor. Thus, the conductivity of the NaCl solution ($\sigma_{l}$) was calculated as:

(14) $$\sigma_{l}=\frac{l}{R_{l}S}$$

where $R_{l}$ was the resistance of the measured NaCl sample, l was the distance between two pin electrodes, and S was the cross-section of the current path, which was the area of copper plates (1000 mm^2^). The measurement was repeated eight times with different distance $l_{i}$ from 5 to 40 mm with 5 mm each step. Then, the mean conductivity and standard deviation (SD) were calculated by:

(15) $$\bar{\sigma_{l}}=\frac{1}{n}\overset{n}{\underset{i=1}{\sum }}\sigma_{l}{}_{i}=\frac{\frac{l_{1}}{R_{l}{}_{1}A}+\frac{l_{2}}{R_{l}{}_{2}A}+\ldots +\frac{l_{n}}{R_{l}{}_{n}A}}{n}$$

(16) $$SD=\sqrt{\frac{\sum_{i=1}^{n}{\left(\sigma_{l}{}_{i}-\bar{\sigma_{l}}\right)}^{2}}{n-1}}$$

Table 2 illustrates the calculated conductivity (mean value and SD) of different NaCl samples from 1 kHz to 1 MHz. The conductivity of salt solution showed a positive relationship with NaCl concentration as in the literature. Although the conductivity slightly increased with the frequency for each sample, the overall values remained relatively constant over the frequency range. Compared with the literature, 0.03 M and 0.15 M samples showed good agreement with previous studies, whereas the measured conductivity of the 0.05 M sample was slightly higher. Based on the linear regression, 0.08 M NaCl solution was selected as the blood simulant in further experiments.

#### 2.3.3. Human Wrist Phantom Fabrication

As shown in Figure 6, there were three main steps for the wrist phantom fabrication:

1. Assemble and seal the printed mould

After assembling all 3D-printed parts and settling the electrode plate at the bottom of the mould, two copper tapes with a width of 10 mm were stuck around the inner wall of the mould and soldered with the wire at one end. Afterwards, all joints between detachable parts were sealed using silicone sealant to prevent the gelatine solution from leaking out.

2. Casting the surrounding tissue simulant

Selected tissue-mimicking materials (20 wt % gelatine powder and 0.1 wt % NaCl) were mixed in deionised water and thoroughly dissolved to fabricate the surrounding tissue simulant. Then, the prepared gelatine solution was gently poured into the mould and left on a perfectly horizontal surface at room temperature for 24 h. After solidifying, the silicone sealant was cleared away, and the metal rod was tenderly pulled out, leaving a uniform, straight hole as the hollow artery. The detachable part 2 was then demounted to uncover the open end of the artery for further pump operation.

3. Connecting the pumping mechanism

During the initial period of the phantom-pumping experiment, we realised that the gelatine-based phantom could achieve a wider deformation range of contraction than expansion. In other words, the closed end of the artery phantom was more likely to burst when we attempted to increase the diameter of the artery section by injecting more volume of blood simulant. Thus, it was decided to withdraw a specific volume of blood simulant from the phantom to decrease the arterial diameter. To achieve it, a rigid plastic tube was inserted into the hollow artery section around 10 mm, which had a slightly bigger outer diameter of 4.5 mm and did not deform during pumping. Super Glue was applied between the open end of the hollow artery phantom and the rigid tube to prevent air from entering the artery system, which was the only type of sealant we found that could firmly bond the plastic tube with a water-based gelatine phantom. Then, the blood simulant was gradually injected into the hollow artery phantom from the bottom of the artery to the open end of the rigid tube by a slender needle. Next, a syringe half-filled with the blood phantom was connected to the open end of the rigid tube. These procedures were sensitively handled to avoid mixing any gas into the artery system.

#### 2.3.4. Phantom Testing Methodology

**Figure 9:**
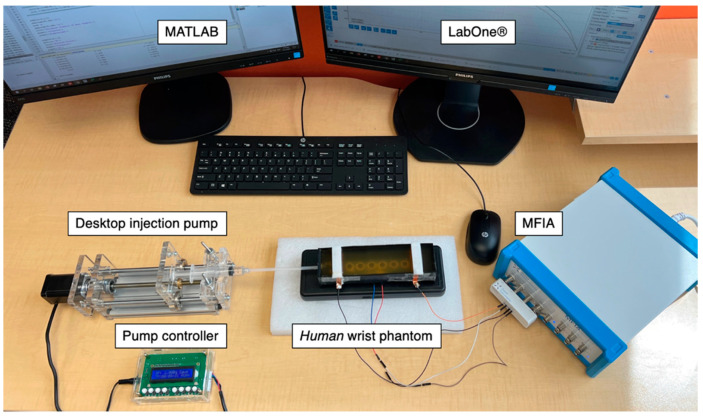
The entire experimental setup for the single artery model phantom-pumping experiments.

Figure 9 represents the integrated experimental setup for impedance measurement during phantom pumping. To ensure stable and controllable operation of the syringe, a commercial desktop injection pump was employed to fix the barrel and drive the plunger, which allowed it to be linearly pulled and pushed along the inside of the barrel with the set speed and time. The MFIA was used to measure the absolute value of the impedance and phase angle of all subjects from 1 kHz to 1 MHz with ten logarithmical steps (i.e., 1 kHz, 2.15 kHz, 4.64 kHz, 10, 21.54 kHz, 46.42 kHz, 100 kHz, 215.44 kHz, 464.16 kHz and 1 MHz) throughout the experiments.

Each electrode was assigned a number to facilitate the description of the different electrode configurations, as shown in Figure 6. Then, electrode configurations that applied conventional four-spot electrodes were assigned codes as ‘L_CC_/L_PU_’, where ‘L_CC_’ indicated the distance between CC electrodes, and ‘L_PU_’ demonstrated the distance between PU electrodes. For those configurations that applied band electrodes, the codes were adjusted as ‘B/L_PU_’, where ‘B’ stood for the band electrode method (see Table 3).

The investigation of phantom pumping was divided into a main experimental group (Group A) and a subgroup (Group B). Each experimental group had specific electrode configurations and pumping operations for different objectives, as shown in Table 4. The original static condition was measured as the basal impedance before contracting the artery in each test. The withdrawn blood simulant was entirely injected back into the artery to allow the arterial diameter to restore to the original geometry for the next test.

#### 2.3.5. Data Processing

In the beginning, the increment volume of the syringe was equal to the decreased blood phantom volume in the artery because of the atmospheric pressure. The reference cross-sectional area changes in the artery ($\Delta A_{r}$) can be calculated from the withdrawn volume of the blood phantom ($\Delta V_{b}$) and the total length of the artery phantom ($L_{artery}$):

(17) $$\Delta A_{r}=\frac{\Delta V_{b}}{L_{artery}}=\frac{\pi \Delta L_{syringe}d_{syringe}{}^{2}}{4\;L_{artery}}$$

where $\Delta L_{syringe}$ was the displacement distance of the plunger and $d_{syringe}$ was the inner diameter of the syringe barrel. Since the initial diameter of the artery phantom ($d_{0}$) was known as 3.98 mm, the reference absolute diameter of the artery ($d_{r}$) could be determined:

(18) $$d_{r}=\frac{A_{0}-\Delta A_{r}}{L_{artery}}=\frac{\frac{\pi d_{0}{}^{2}}{4}-\frac{\Delta L_{syringe}A_{syringe}}{L_{artery}}}{L_{artery}}=\frac{\pi d_{0}{}^{2}}{4L_{artery}}-\frac{\Delta L_{syringe}A_{syringe}}{L_{artery}{}^{2}}$$

For the measured data from MFIA, frequency (f), absolute impedance (|Z|), and phase angle (θ) were exported from the LabOne^®^ control interface and then processed and analysed using MATLAB (R2019a, The MathWorks, Natick, MA, USA). First, the real and imaginary parts of measured impedance were calculated by:

(19) $$Re\left(Z\right)=\left|Z\right|\cos\left(\frac{\pi \theta }{180}\right)$$

(20) $$Im\left(Z\right)=\left|Z\right|\sin\left(\frac{\pi \theta }{180}\right)$$

The electrical response of the wrist phantom was also described using a parallel RC circuit. Thus, the resistance of the whole wrist phantom can be obtained. Afterwards, according to Equations (9) and (10), the diameter of the artery phantom after pumping can be estimated by:

(21) $${\hat{d}}_{artery}=2\sqrt{\frac{{\hat{A}}_{artery}}{\pi }}=2\sqrt{\frac{A_{0}-{\hat{dA}}_{artery}}{\pi }}=2\sqrt{\frac{A_{0}+\frac{L_{PU}\cdot \left(R_{0}-R_{p}\right)}{\bar{\sigma_{l}}\cdot R_{0}\cdot R_{p}}}{\pi }}$$

where $L_{PU}$ was the distance between two pick-up ECG dry electrodes, $R_{0}$ was the basal resistance of the whole wrist phantom corresponded with the original cross-sectional area of the artery phantom ($A_{0}=\frac{\pi d_{0}{}^{2}}{4}\approx 12.44\;mm^{2}$), $R_{p}$ was the measured resistance of the whole wrist phantom after pumping, and $\bar{\sigma_{l}}$ was the mean conductivity of the blood simulant (0.08 M NaCl) calculated by Equation (15). The percent error ($PE_{artery}$) was calculated to demonstrate the accuracy of the estimated arterial diameter:

(22) $$PE_{artery}=\frac{\left|d_{r}-{\hat{d}}_{artery}\right|}{d_{r}}\times 100\%$$

## 3. Results

### 3.1. Computational Simulation

#### 3.1.1. Current Density Distribution and Electric Field

**Figure 10:**
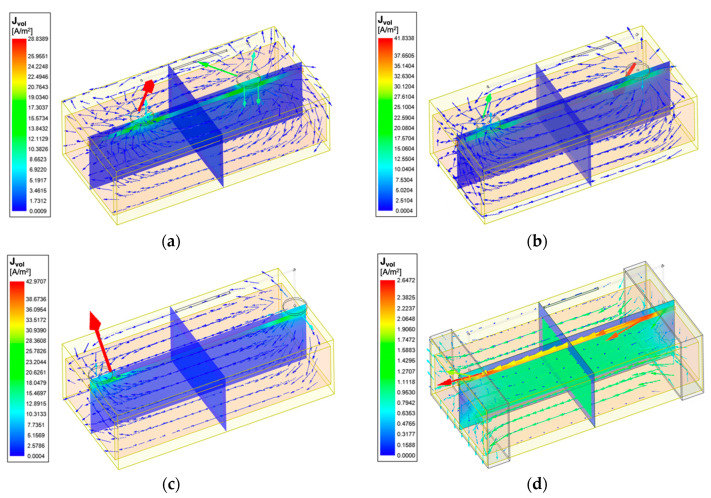
Current vectors and current density overlay of the wrist model 1 at 100 kHz: (**a**) 4-spot electrodes with $L_{CC}$ = 50 mm; (**b**) 4-spot electrodes with $L_{CC}$ = 70 mm; (**c**) 4-spot electrodes with $L_{CC}$ = 90 mm; (**d**) band electrodes with $L_{CC}$ = 90 mm.

**Figure 11:**
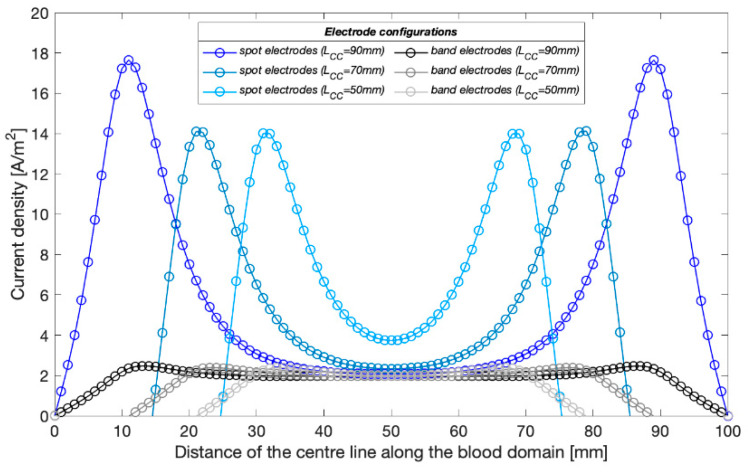
Current density distribution along the blood-filled artery centre of the wrist model 1 with different CC electrodes configuration at 100 kHz.

Figure 10 shows the 3D current density distribution for different simulation setups at 100 kHz in wrist model 1. Additionally, the current density curves along the blood have been plotted in Figure 11. The spot electrodes setup with the $L_{CC}$ of 50 mm produced the smallest region of parallel current density along the artery. $L_{CC}$ of 90 mm showed a nearly uniform distribution, specifically between 40 and 60 mm. For band electrodes, $L_{CC}$ of 50 mm still produced the least parallel current density; however, it was much larger than the spot electrodes. $L_{CC}$ of 90 mm with band electrodes produced the most uniform field, specifically between 20 and 80 mm.

**Figure 12:**
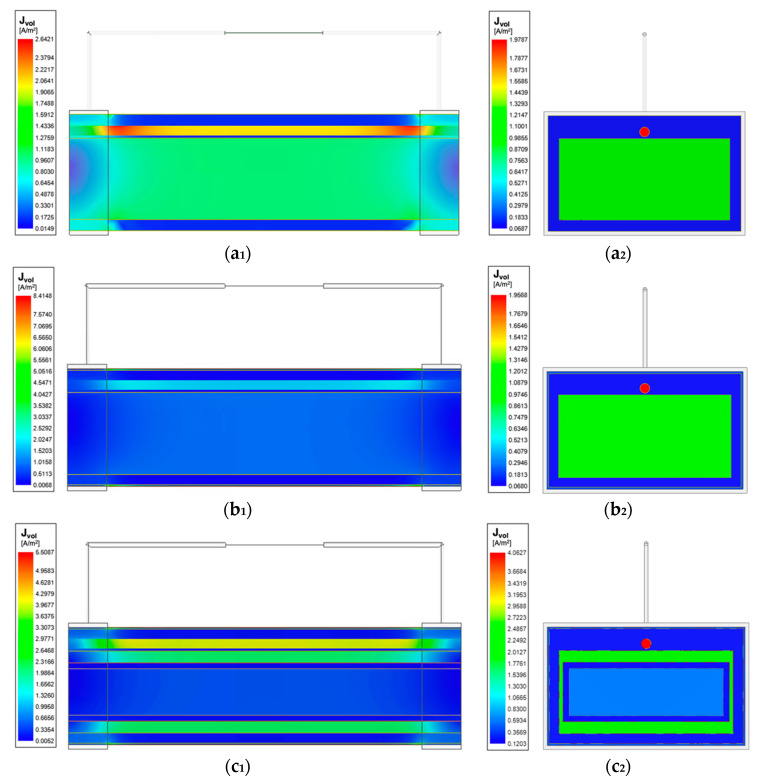
Current density overlay of stepwise-updated wrist models at 100 kHz: (**a_1_**) horizontal cross-section of the wrist model 1; (**a_2_**) vertical cross-section of the wrist model 1; (**b_1_**) horizontal cross-section of the wrist model 2; (**b_2_**) vertical cross-section of the wrist model 2; (**c_1_**) horizontal cross-section of the wrist model 3; (**c_2_**) vertical cross-section of the wrist model 3.

The initial investigation in wrist model 1 showed the reliability of using band electrodes over spot electrodes due to the more uniform field distribution. As such, the following investigation included the addition of other tissues with the band electrode configuration. The impact of including different tissues in the three models, each of which exhibit a different conductivity spectrum in the β-dispersion frequency range, resulted in different amplitudes and extents of the current density distribution, which has been shown in Figure 12. In all the three models, the blood domain had the maximum current density compared with other tissues, which was followed by the muscle domain.

#### 3.1.2. Simulated Basal Resistance

**Figure 13:**
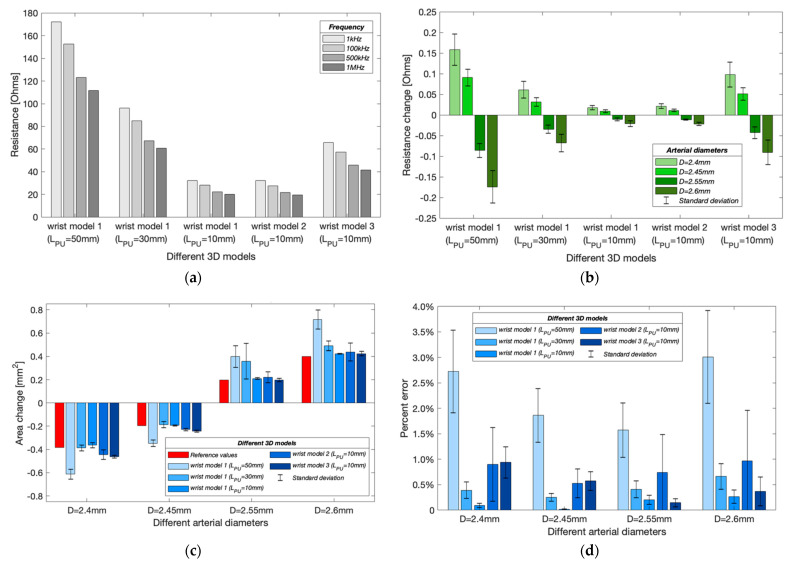
Simulation results in different wrist models with band electrodes configurations: (**a**) simulated basal resistance at different frequencies; (**b**) simulated resistance changes caused by arterial diameter changes (mean ± SD); (**c**) estimated cross-sectional area changes of the artery (mean ± SD); (**d**) percent errors of estimated arterial diameters (mean ± SD).

Following the initial current density distribution investigation, resistance was calculated by taking the line integral of electric field between the PU electrodes. At this stage, the basal resistance ($R_{0}$) for band electrode configuration was calculated for three different spacings (50 mm, 30 mm and 10 mm) of PU electrodes ($L_{PU}$) in the wrist model 1. In any model, the setup with an artery diameter of 2.5 mm was defined as the basal condition. As shown in Figure 13a, with an $L_{CC}$ of 90 mm, $R_{0}$ for an $L_{PU}$ of 50 mm and 30 mm were approximately five times and three times larger than that for an $L_{PU}$ of 10 mm, respectively, thus exhibiting a nearly proportional relationship with $L_{PU}$. The basal resistances reduced with the increasing frequency in all models, corresponding to the frequency-dependent tissue conductivities. For the modified wrist model with the added skin layer (wrist model 2), the simulated resistances were slightly lower at 100 kHz, 500 kHz and 1 MHz because of the higher conductivity of skin than fat tissue. On the contrary, the simulated resistance was slightly greater than the wrist model 1 at 1 kHz, which was due to its lowest conductivity (0.00065738 S/m). In the wrist model 3, the high resistance of bones significantly increased the simulated resistance by around 20 to 30 Ω across the frequency range.

#### 3.1.3. Simulated Resistance Changes with Different Arterial Diameters

In the simulation model, the expansion and contraction of the radial artery were represented by different diameter instances such as 2.6 mm, 2.55 mm and 2.45 mm, 2.40 mm. Results from Figure 13b validate the contribution of blood volume change on the overall measured impedance due to the higher conductivity of the blood than other tissues. As the arterial diameter increased, the simulated resistance decreased and vice versa. For wrist model 1, the resistance decreased with decreasing $L_{PU}$. However, the resistances after the addition of a skin layer did not show significant differences from the initial wrist model. Differently, after adding bones, the changing range of simulated resistances was about four times greater than model 1. Moreover, the simulated resistance change decreased with increasing frequency for all wrist models.

#### 3.1.4. Estimated Arterial Diameters

Figure 13c shows the estimated cross-sectional area changes at different arterial diameters. The red bars indicate the target (reference) values of the cross-sectional area change. For wrist model 1, a shorter $L_{PU}$ could achieve closer estimation, corresponding to the more uniform E-field in the middle region. Figure 13d shows the mean percent errors of the estimated arterial diameters. The shortest $L_{PU}$ showed the lowest error due to a more uniform measurement region. For an arterial diameter of 2.6 mm, the errors reduced to 0.26 ± 0.13% from 2.72 ± 0.81% when $L_{PU}$ was decreased to 10 mm from 50 mm. However, it was found that the percent errors increased after adding other tissue domains (wrist models 2 and 3). For an arterial diameter of 2.6 mm, the error increased to 0.96 ± 0.90% and 0.36 ± 0.28% in the wrist models 2 and 3, respectively. The frequency-dependent errors were also noticed, where the simulation at 1 kHz usually obtained the highest accuracy, and the results at 500 kHz and 1 MHz were found to be worse. Furthermore, a diameter-dependent error was also found. The estimated arterial diameters were usually more accurate when estimating relatively smaller area changes i.e., 2.45 mm and 2.55 mm.

### 3.2. Tissue Phantom Experiment

#### 3.2.1. The Properties of Selected Simulants

The mechanical properties of the surrounding tissue simulant principally depended on the amount of gelatine. Its strength was likely to increase with a longer storage time, as observed before [35]. During the entire experiment, the 20 wt % gelatine phantom showed an adequate rupture strength to maintain the integrity of the artery during pumping without any breaking.

**Figure 14:**
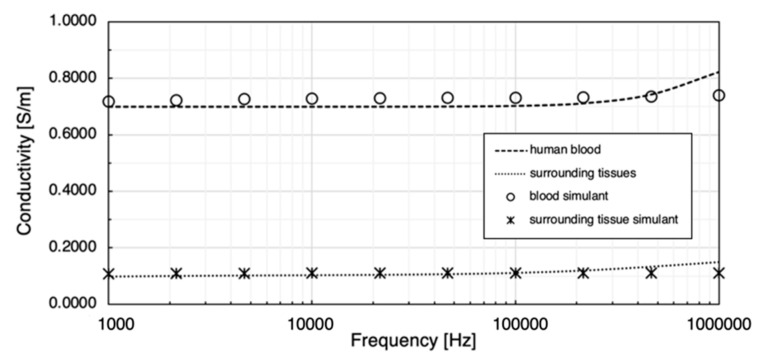
Comparison between prepared tissue simulants and target conductivities.

In this work, 0.08 M NaCl solution was prepared as the human blood simulant and 20 wt % gelatine powder and 0.1 wt % NaCl were dissolved in deionised water to simulate the overall surrounding tissues. Their bulk conductivities at room temperature were determined using MFIA and plotted in Figure 14. Both simulants showed similar ranges of conductivity with the target values. The conductivity (mean ± SD) of 0.08 M NaCl solution was from 0.7171 ± 0.0178 S/m to 0.7395 ± 0.0172 S/m between 1 kHz and 1 MHz. The conductivity of the surrounding tissue simulant was between 0.1085 and 0.1112 S/m in the interested frequency range.

#### 3.2.2. Group A: Different Electrode Configurations

**Figure 15:**
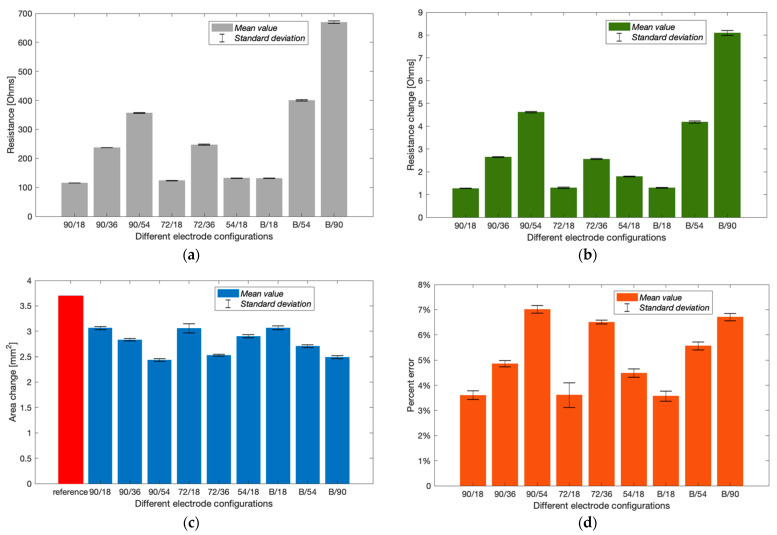
Experimental results of Group A (mean ± SD): (**a**) measured basal resistance; (**b**) measured resistance change after pumping; (**c**) estimated cross-sectional area change; (**d**) calculated percent errors of the estimated arterial diameter.

The initial phantom-pumping test was performed with six different conventional four-spot electrode configurations and three band electrode configurations. The basal resistance of the wrist phantom ($R_{0}$) was calculated via measured absolute impedance and phase angle, as shown in Figure 15a. With the same CC electrode spacing ($L_{CC}$ = 90 mm), the resistance measured by the longest $L_{PU}$ of 54 mm (356.58 ± 1.14 Ω) and medium $L_{PU}$ of 36 mm (237.38 ± 0.34 Ω) were approximately 3.10 times and 2.06 times larger than the shortest $L_{PU}$ of 18 mm (114.97 ± 0.19 Ω), which also showed a fairly proportional relationship with the $L_{PU}$, as observed in the simulation section. The same phenomenon was found by comparing 72/18 (123.49 ± 0.73 Ω) and 72/36 (247.05 ± 1.69 Ω) electrode configurations. The band electrodes were constructed using two copper tapes stuck around the wrist phantom with a longer $L_{CC}$ of 126 mm. The nearly proportional relationship between $L_{PU}$ and $R_{0}$ was indicated in band electrode configurations as well. The basal resistance values of B/90 (669.80 ± 4.63 Ω) and B/54 (400.43 ± 2.43 Ω) were 5.10 times and 3.05 times greater than B/18 (131.29 ± 0.86 Ω). Compared to the influence of $L_{PU}$, $L_{CC}$ only exhibited tiny impacts on the measured resistances.

Figure 15b shows the increment in resistance after withdrawing 647.83 mm^3^ blood simulant. After withdrawing the blood simulant, the increased volume in the barrel was equal to the decreased volume in the artery phantom. Thus, the reference cross-sectional area change of the blood simulant and arterial diameter was 3.7019 mm^2^ and 3.34 mm, respectively. From Figure 15c, all electrode configurations were likely to underestimate the area change. Configurations 90/18, 72/18, 54/18 and B/18 showed relatively closer estimation than others. According to Figure 15d, the most accurate estimation was obtained by configuration B/18 with the lowest percent error of 3.57 ± 0.19%, which was followed by 90/18 (3.60 ± 0.17%) and 72/18 (3.61 ± 0.49%). It was worth noting that the configuration 72/18 exhibited a larger SD due to significant shifts at the last two frequencies. Configurations B/18 and 90/18 showed slight differences in basal resistance, resistance change and even the estimated arterial diameter. In other words, the band electrode method did not exhibit superior performance to the four-spot electrodes method for the least $L_{PU}$.

#### 3.2.3. Group B: Potential Sources of Errors

This experimental phase was an extension of Group A intended to explore the potential source of errors. We re-tested the band electrode configuration B/18. Furthermore, the pumping process was finely divided into ten minor constrictions of 80.98 mm^3^ blood simulant per step.

**Figure 16:**
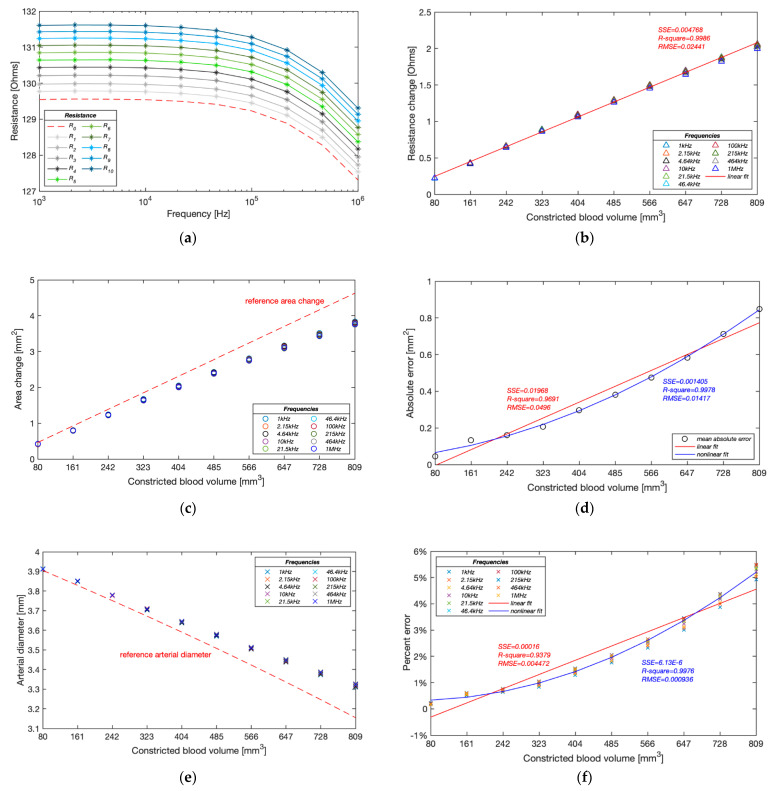
Experimental results of Group B: (**a**) measured resistance with ten pumping times; (**b**) measured resistance change during pumping; (**c**) estimated cross-sectional area change of the artery during pumping; (**d**) average absolute errors of estimated cross-sectional area change during pumping; (**e**) estimated arterial diameter during pumping; (**f**) relative errors of estimated arterial diameters.

As shown in Figure 16a, the basal resistance of the wrist phantom decreased from 129.55 Ω to 127.31 Ω between 1 kHz and 1 MHz, which was due to the slightly increasing conductivity of the gelatine-base phantom. The measured resistance raised progressively with each equal amount of blood simulant withdrawn and achieved 131.61 Ω to 129.31 Ω after the last contraction. The linear relationship between the increment in resistance and the constricted volume of blood simulant has been indicated by the linear regression in Figure 16b. The average incremental resistance per constriction was 0.204 ± 0.017 Ω, whereas a slight decreasing trend of the increment was found from the first constriction to the last constriction, which might be one potential measurement error.

Figure 16c,d demonstrate the accuracy of the estimated cross-sectional area change of the artery phantom. Different from the simulation results, the proposed method was likely to underestimate the blood simulant volume change in the experiments. A significant deviation from the reference values was noticed after the fifth contraction (404.90 mm^3^) and then became more deflective with the incremental constricted volume of blood simulant. The absolute error reached 0.84 mm^2^ after the last contraction. The absolute errors of estimated cross-sectional area change showed a more reliable non-linear fit (quadratic polynomial regression) than the linear fit, exposing a non-linear relationship with the blood volume change.

The deviation of the estimated area change led to a similar underestimation in diameter, as shown in Figure 16e. The average deviation of the estimated diameter was only 0.007 mm after the first contraction and then gradually expanded to 0.167 mm in the end. Moreover, the estimated values at different frequencies were pretty consistent at the beginning of pumping, while the differences became significant as the artery continued to be constricted. Figure 16f shows that the lower frequencies usually obtained higher accurate estimation. The correlation between the percent errors and constricted blood volume showed a non-linear relationship (quadratic polynomial regression) rather than a linear relationship (first-degree polynomial regression), which demonstrated that the proposed method was able to sensitively track the volume change of the blood and thereby accurately estimate the arterial diameter in a certain deformation range. In the computational simulations, the arterial diameter was modelled between 2.4 and 2.6 mm based on the actual vasoconstriction and vasodilation range of the human radial artery. The minimum arterial diameter was 92.3% of the maximum arterial diameter. According to this ratio, the equivalent range of artery phantom was assumed between 3.67 and 3.97 mm, corresponding to the fourth pumping in this experimental group. The band electrode configuration *B/18* and proposed mathematical modelling within this deformation range was found to achieve a pretty accurate estimation with a percent error of 0.98 ± 0.07%.

## 4. Discussion

### 4.1. The Contribution of Different Tissues to Impedance Measurement

In the simulation section, this paper attempted to improve the understanding of different responses and contributions of each tissue under BIM and then provide evidence to model tissue domains in future simulations felicitously.

The current density overlays (see Figure 10 and Figure 12) determine that the amounts of flowing current were decided by the conductivity of tissues. As blood had the highest conductivity, more current flowed into the arteries, which was followed by the muscle. Skin shows low conductivity at 1 kHz (100 times lower than fat) and then gradually becomes more conductive as the frequency increases, surpassing the fat and cortical bone at 50 kHz and exceeding the cancellous bone at 200 kHz. Bones, as the major component of the human wrist, had relatively lower conductivity across the frequency range of interest. The added bone domains accounted for about 68% of the original muscle domain, causing larger simulated basal resistance. As a result, more amounts of injected current flowed into the conductive regions such as the blood and muscle domains, inducing higher resistance change caused by arterial diameter change. Therefore, for future investigations that want to focus on the contribution of pulsatile blood, it is recommended to include the bones in the human 3D model to highlight the contribution of conductive blood. Even though lower frequencies obtained higher simulated resistance change, it worth noting the electrode–skin contact impedance is also normally more significant at the low-frequency range.

### 4.2. The Effects of Electrode Spacings

The highlight of this work has been the investigation’s focus on the effect of different electrode spacings—$L_{CC}$ and $L_{PU}$. Initially, the simulations compared the uniformity of current density in the wrist model 1 with three CC electrode spacings of the four-spot electrodes method. Figure 11 demonstrates that the current density in the middle region between the CC electrodes was approximately uniform by increasing the CC electrode spacing. A shorter spacing meant that the overall system could take up less space, which can be more portable and inconspicuous in actual measurement. At the next stage of simulation, an $L_{CC}$ of 90 mm was chosen for both the four-spot electrodes method and the band electrode method. Three PU electrode spacings ($L_{PU}$ = 50 mm, 30 mm, and 10 mm) showed a nearly proportional relationship between simulated basal resistance of the wrist model 1 and $L_{PU}$. Moreover, a larger $L_{PU}$ was found more sensitive to changes caused by arterial blood volume change and hence indicative of obtaining more pronounced pulse waves in the actual measurement. However, it did not lead to a more accurate estimation of arterial diameters. Conversely, shorter $L_{PU}$ values induced lower percent errors of estimated diameters because shorter $L_{PU}$ values comprised a more uniform E-field region.

In order to verify the simulated outcomes, three CC electrode spacings and three PU electrode spacings were separately measured in the phantom experiments: $L_{CC}$ values of 90 mm, 72 mm, and 54 mm and $L_{PU}$ values of 541 mm, 36 mm, and 18 mm. The phantom experimental results were consistent with simulation results. The nearly proportional relationship between the measured resistance of the wrist phantom and the $L_{PU}$ was also indicated in all experimental tests. Furthermore, shorter $L_{CC}$ induced slightly higher measured resistance for the same $L_{PU}$. The amounts of measured resistance and resistance changes were mostly sensitive to $L_{PU}$ than $L_{CC}$.

### 4.3. The Effects of Electrode Types

The proposed band electrode showed better performance than the conventional four-spot electrode approach in the simulation due to a larger extent of parallel E-field distribution within the model. Even though adding tissue components weakened the estimation of arterial diameter in the wrist model 3, the band electrode configuration still achieved an average percent error of less than 1.00%.

In the phantom experiments, for the same $L_{PU}$, band electrode configurations appeared to have better estimations than the conventional four-spot electrode configurations. We believe that band electrodes are also highly implementable in the actual BIM, which can be performed by the wristband made of types of conductive textiles. Even though the conventional Ag/AgCl electrode has stable adhesion on the skin and lower skin–electrode impedance, the degradation of electrolyte gel with time can reduce its durability for long-term use. The textile fabrication of electrodes provides more flexibility and wearability for long-term monitoring by avoiding the use of gels. In recent years, particular attention has been focused on developing textile electrodes for types of hemodynamic monitoring [39,40,41,42,43]. Thereby, it is predictable that the textile electrodes will play a promising role in ambulatory hemodynamic monitoring.

### 4.4. Potential Sources of Errors

The deformation range of the artery was modelled between 2.4 and 2.6 mm based on the actual vasoconstriction and vasodilation range of the human radial artery in the simulation, scaling up from 3.67 to 3.97 mm in the phantom experiments. Within this range, the band electrode configuration was found to achieve high accuracy in the estimation of arterial diameter with a percent error of less than 1.00% in both simulations ($L_{CC}$ = 90 mm, $L_{PU}$ = 10 mm) and phantom experiments ($L_{CC}$ = 126 mm, $L_{PU}$ = 18 mm).

The diameter-dependent error was noticed in the simulation; the larger diameter changes always induced the larger percent error. The primary source of this error was the inherent difference between the simulation condition and the theoretical condition. The proposed mathematical model assumed that the pulsation led to an additional volume change in the original geometry. However, in the simulation setup, the artery diameter change was implemented by re-assigning the surrounding region of the artery, which means the overall volume was kept the same as the original geometry. Therefore, larger diameter variation caused a more significant estimation error.

For phantom experimentation (especially group B), the following potential sources of errors might have affected the final accuracy of the estimation:

1. The increased errors in the larger deformation range were due to the reduced reliability of the mathematical model, which assumed a parallel flow of current with blood.
2. Higher frequencies usually showed poor estimation due to distinct electrical responses of the wrist phantom to different frequencies and possible measurement errors.
3. The conductivity of the gelatine-based phantom was sensitive to temperature and moisture content, which might have impacted the impedance reading.
4. The effects of electrode–phantom surface polarisation from the measured impedance were not eliminated; thereby, a higher ‘measured’ resistance led to under-estimating the cross-sectional area change, which is also desired to be eliminated in the actual BIM with human body.
5. The reference arterial diameters were also calculated instead of measured. The unnoticed presence of air in the artery-pump system or any faulty sealing at the junction could lead to an erroneous calculation of cross-sectional area change.

### 4.5. Limitations and Future Works

Even though this work accomplished promising accuracies of estimated arterial diameters, it worth noting that the results in both simulation and phantom experiments were based on a few assumptions (e.g., constant tissue properties, simplified geometry, etc.). Perceiving the distinction between simulation/phantom and the actual anatomy of the human wrist, several potential limitations need to be considered, and more efforts are desired to be addressed in the future.

Firstly, the mathematical modelling relied on the uniformity of the E-field inside the measured body segment. However, the real properties of human tissues are not homogenous, and the anatomy is much more complex (e.g., smaller arteries and arterioles). Human tissues as dielectric materials also exhibit distinct frequency-dependent permittivity. Future modelling could take the permittivity into account to reveal more dielectric responses in the frequency range. Additionally, beyond the RC circuit modelled in this work, other equivalent circuits for human tissue can be applied and further explored, such as [44,45]. Furthermore, different from the present simulation and phantom situations, knowledge of absolute arterial diameters required an initial calibration in the actual measurement, requesting another technique to determine the basal diameter, such as the ultrasound. Several previous studies already investigated and provided promising ultrasound-based techniques and systems for accessorial arterial diameter measurement [46,47]. The arterial diameter change shows a strong relationship with BP change, which is expected to be further estimated by calibrating the basal BP rather than the basal diameter.

Secondly, from a numerical simulation perspective, the 3D wrist model could be developed further. This simulation only represented the pulsatile blood as several instances of arterial diameters. Future research is expected to model blood flow dynamics and simultaneously observe impedance changes, assigning the velocity-dependent blood conductivity change to represent contribution of RBC orientation. Moreover, the geometry can be refined to be more realistic, separating the bone domain into individual radius and ulna, and gradually changing the thickness of tissue layers from distal to proximal.

Lastly, the representation of blood flow in the forearm has been implemented through a single (radial) artery in this work, and the band electrode configuration exhibited the promising capability to sense the overall blood volume change throughout the measured segment. While the objective here was to investigate the measurement of effective diameter changes due to overall blood flow, future work may be directed towards exploring and possibly isolating the response of two major arteries (i.e., radial artery and ulnar artery) and other smaller vessels in the forearm.

## 5. Conclusions

This work attended to improve the accuracy of quantification in the arterial diameter-dependent impedance variance by altering the electrode configuration from computational simulation and artificial tissue phantom perspectives. The blood volume change was assumed as the main contribution on impedance changes in this work. An FEM was carried out with human wrist fragment using ANSYS HFSS, helping in understanding the dielectric response of multi-tissues and blood flow from 1 kHz to 1 MHz, the current distribution throughout the wrist region, and the optimisation of electrode configurations for arterial pulse sensing. Then, a corresponding wrist phantom was fabricated using non-toxic and low-cost materials. The blood-filled artery was constricted using a desktop injection pump, and the impedance change was measured by the MFIA. The simulation revealed the promising capabilities of band electrodes to generate a more uniform current distribution than the traditional spot electrodes. Both simulation and phantom experimental results indicated that a longer spacing between CC electrodes with shorter spacing between PU electrodes in the middle could sense a more uniform E-field, engendering a more accurate arterial diameter estimation.

## Figures and Tables

**Table 1:** Stages for computational simulation.

Stages	3D Models	Tissue Domains					Current Carrying Electrodes (Platinum)
		Fat	Muscle	Blood	Skin	Bones	
1	Wrist model 1	✓	✓	✓			Spot electrode $(L_{CC}$ = 90 mm, 70 mm and 50 mm) Band electrode $(L_{CC}$ = 90 mm, 70 mm and 50 mm)
2	Wrist model 2	✓	✓	✓	✓		Band electrode $(L_{CC}$ = 90 mm)
3	Wrist model 3	✓	✓	✓	✓	✓	Band electrode $(L_{CC}$ = 90 mm)

**Table 2:** Comparison between measured mean conductivity and reference values from the literature.

NaCl Samples	$\mathrm{Conductivity}\;\bar{\sigma_{l}}$ (S/m)(Mean ± SD)	Reference Values	
		Gabriel et al., 2009 [37] (Four Electrodes)	Peyman et al., 2007 [38] (Open-Ended Coaxial Probe)
0.03 M	0.270 ± 0.0017	0.276	0.281
0.05 M	0.514 ± 0.0071	0.477	0.466
0.15 M	1.374 ± 0.0280	1.392	1.375

**Table 3:** Assigned codes for various electrode configurations in phantom pumping experiments.

Electrode Methods	Electrode Configurations						
	Locations of CC Electrodes		Spacing between CC Electrodes	Locations of PU Electrodes		Spacing between PU Electrodes	Assigned Codes
Conventional 4-spot electrodes method	1	6	90 mm	3	4	18 mm	90/18
	1	6	90 mm	2	4	36 mm	90/36
	1	6	90 mm	2	5	54 mm	90/54
	1	5	72 mm	3	4	18 mm	72/18
	1	5	72 mm	2	4	36 mm	72/36
	1	4	54 mm	2	3	18 mm	54/18
Band electrodes method	Band electrodes		126 mm	1	6	90 mm	B/90
	Band electrodes		126 mm	2	5	54 mm	B/54
	Band electrodes		126 mm	3	4	18 mm	B/18

**Table 4:** Experimental details of each test group.

Test Groups	Pump Times	Applied Electrode Configurations			Initial Arterial Diameter (*d*_0_)	The Volume of Constricted Blood (Δ*V*)	Reference Arterial Diameter (*d_r_*)
A	1	90/18 90/36 90/54	72/18 72/36 54/18	B/18 B/54 B/90	3.98 mm	647.83 mm^3^	3.34 mm
B	1	B/18				80.98 mm^3^	3.91 mm
	2					161.96 mm^3^	3.83 mm
	3					242.94 mm^3^	3.75 mm
	4					323.92 mm^3^	3.67 mm
	5					404.90 mm^3^	3.59 mm
	6					485.88 mm^3^	3.51 mm
	7					566.86 mm^3^	3.42 mm
	8					647.84 mm^3^	3.34 mm
	9					728.82 mm^3^	3.25 mm
	10					809.80 mm^3^	3.15 mm

## Data Availability

Not applicable.
